# Medical journalology and questionable research practices

**DOI:** 10.3399/bjgp25X742485

**Published:** 2024-05-30

**Authors:** Euan Lawson

**Affiliations:** Lancaster University, Lancaster.

The *BJGP* is the world’s leading journal in primary care and general practice. I’m happy to defend this assertion but the temptation is to reach for a metric to support the case. And of course, the obvious number to which one turns is the infamous and utterly unavoidable impact factor.^1^ The impact factor is calculated per journal and is simply the total number of citations in the current year divided by the number of papers published in the two years before that. It is childishly basic yet it has a gargantuan effect on behaviour.

In itself the impact factor is not inherently wrong but it is just so limited and it is an ugly shorthand for assessing the worth of journals and of researchers. Yet it ploughs on, unkillable, a lone zombie metric. The *BJGP* is a signatory to the San Francisco Declaration of Research Assessment (DORA)^2^ and as part of that, one of the elements we sign up to is that we won’t use the impact factor, in isolation, to promote the journal.

Our role at the *BJGP* is not to compete against other journals but to publish primary care research for patients, clinicians, and policymakers. And we want to do it with as much integrity as we can muster in a system that is flawed in many ways. Journals can trumpet their impact factors to the skies but at its heart it is a one-dimensional approach with alarming unintended consequences. Academics are too often compelled to seek out ‘high impact factor’ journals irrespective of other considerations. Impact factors are one strand in what can be a toxic tapestry of ‘publish or perish’ academic norms which lead to concerns around research integrity.

## Research integrity, the replication crisis, and QRPs

As concerns about the integrity of science have emerged so the field of meta-science has grown. The replication crisis, which can be traced back to the 2010s, has been acutely felt in fields such as psychology.^3^ The replication crisis has been the realisation that many of the studies that have been widely disseminated, and accepted as fact, have been discovered to be unreplicable. In other words, they’re almost certainly not reliable evidence. This is closely linked to a reproducibility crisis. Some of the root causes are well known and pithily described by Dorothy Bishop as the four horsemen of irreproducibility: low statistical power, *P*-hacking, HARKing, and publication bias.^4^

Box 1 **Key terms in metascience**

**Table:** 

Reproducibility: to reanalyse data and get the same results.
Replicability: to repeating the same research with a new dataset and get the same findings
Low statistical power: studies with too few participants to achieve reliable results and prone to type 1 errors.
*P*-hacking: Manipulating data, deliberately or inadvertently, to achieve statistical significance (usually *p*<0.05).
HARKing: Hypothesising After the Results are Known. A process of data dredging where results are combed for statistically significant findings.
Publication bias: the tendency for journals to accept papers with positive and/or novel results.

Meta-science is emerging as an increasingly important discipline and the UK Metascience Unit was created in 2024. It is about recognising the power of the scientific method and turning it inwards; they assert that science must be 'systematically and routinely applied to how we practice, fund, and support science itself.'^5^ Questionable research practices (QRPs) have come to prominence as meta-scientists have explored the potential challenges of addressing the root causes that may compromise the integrity of science. A recent paper in PLOS laid out over 20 QRPs and discovered when they surveyed researchers over five countries, including Denmark, UK, USA, Austria, and Croatia, that QRPs are widespread.^6^ It’s helpful to think of QRPs as a spectrum ranging from really very minor, inadvertent glitches in the research process, to outright deliberate, unethical and fraudulent practice. Table 1 outlines some self-reported practices that have been labelled as QRPs and were explored in that survey — these are not rare events with 38–65% of researchers reporting recent use of various QRPs.

**Table 1:** Common self-reported questionable research practices and their incidence (QRPs). Adapted from Schneider *et al*.^6^

	Self-reported in recent publications Danish participants *n*/*N* (%)	Self-reported in recent publications International participants *n*/*N* (%)
Selective over-citing of own publications	1057/1649 (64)	436/674 (65)
Cite literature without reading it	934/1573 (59)	401/663 (60)
Cite irrelevant literature to please	1001/1688 (59)	427/688 (62)
Lack of sufficient effort when reviewing	230/427 (54)	86/179 (48)
Report non-significant findings as evidence of no effect	382/722 (53)	151/274 (55)
Do not distinguish between statistical and practical significance	375/720 (52)	121/253 (48)
Honorary authorships	412/841 (49)	165/364 (45)
HARKing in confirmatory quantitative studies	755/1614 (47)	286/580 (49)
Overselling results	718/1604 (45)	321/575 (56)
Salami-slicing publications	502/1135 (44)	172/373 (46)
Cherry-pick what supports a hypothesis and disregard what does not	639/1563 (64)	271/567 (48)
Deliberately publishing redundant work	650/1608 (40)	294/651 (45)
Undisclosed data-dredging – *p*-hacking	656/1710 (38)	269/626 (43)

## Applicability to primary care

It might be tempting in the field of primary care research to feel that perhaps it doesn’t apply. We’re certainly fortunate at the *BJGP* to receive submissions from a committed primary care research academic workforce but there is no area of scientific endeavour that will not be affected by QRPs.

It is possible to frame QRPs as a positive influence and a roadmap to higher quality primary care research. When talking about them, we must be careful to avoid the suggestion that researchers and research departments are pursuing selfish policies of questionable practice. Yet it’s incredibly important we can recognise the ways in which research can just slip away from the ideal. At the *BJGP* when we look at the common QRPs, it is apparent that the policies and behaviours of medical journals matter. We are too often complicit in these gently subversive influences on research integrity.

## Journalology

There are many aspects of metascience which are specifically linked to journals and could be filed under the heading ‘journalology’. We have made and we will continue to make changes with these in mind. These include how we manage the peer review process and how we assess competing interests. Journals have a role to play in publication bias and we are mindful of our responsibilities. We want to give authors sufficient words to communicate the science they need. We will continue to encourage open data arrangements and we actively mandate pre-registration protocols. We will continue to scrutinise competing interests and ask hard questions about authorship. The use of pre-registration protocols can go a long way to managing some of the challenges around processes such as HARKing — we have, for many years, offered the innovative option of Registered Reports, though uptake has been low.

We won’t be talking about the impact factor at the *BJGP* but we do want to talk to you about how we can help clinicians and academics to maintain the highest standards in primary care research. This must be the direction of travel for the *BJGP* — a non-profit, ethical publisher of science, that is part of a community seeking to improve research integrity, a cornerstone of clinical practice and healthcare policy. Now, that is world-leading, though we accept we will never be able to pin a number on it.
